# Isolation and genetic characterization of MERS-CoV from dromedary camels in the United Arab Emirates

**DOI:** 10.3389/fvets.2023.1182165

**Published:** 2023-08-31

**Authors:** Abdelmalik Ibrahim Khalafalla, Hassan Zackaria Ali Ishag, Hamdoon Ismail Abdulla Albalushi, Zulaikha Mohamed Abdel-Hameed Al-Hammadi, Saeed Mohamed Saeed Al Yammahi, Asma Abdi Mohamed Shah, Salama Suhail Mohammed Al Muhairi

**Affiliations:** Biosecurity Affairs Division, Development and Innovation Sector, Abu Dhabi Agriculture and Food Safety Authority (ADAFSA), Abu Dhabi, United Arab Emirates

**Keywords:** MERS-CoV, coronavirus, isolation, characterization, dromedary, camel, zoonotic disease

## Abstract

**Background:**

The study of coronaviruses has grown significantly in recent years.

Middle East respiratory syndrome coronavirus (MERS-CoV) replicates in various cell types, and quick development has been made of assays for its growth and quantification. However, only a few viral isolates are now available for investigation with full characterization. The current study aimed to isolate MERS-CoV from nasal swabs of dromedary camels and molecularly analyze the virus in order to detect strain-specific mutations and ascertain lineage classification.

**Methods:**

We isolated the virus in Vero cells and adapted it for *in vitro* cultivation. The isolates were subjected to complete genome sequencing using next-generation sequencing followed by phylogenetic, mutation, and recombination analysis of the sequences.

**Results:**

A total of five viral isolates were obtained in Vero cells and adapted to *in vitro* cultures. Phylogenetic analysis classified all the isolates within clade B3. Four isolates clustered close to the MERS-CoV isolate camel/KFU-HKU-I/2017 (GenBank ID: MN758606.1) with nucleotide identity 99.90–99.91%. The later isolate clustered close to the MERS-CoV isolate Al-Hasa-SA2407/2016 (GenBank ID: MN654975.1) with a sequence identity of 99.86%. Furthermore, the isolates contained several amino acids substitutions in ORF1a (32), ORF1ab (25), S (2), ORF3 (4), ORF4b (4), M (3), ORF8b (1), and the N protein (1). The analysis further identified a recombination event in one of the reported sequences (OQ423284/MERS-CoV/dromedary/UAE-Al Ain/13/2016).

**Conclusion:**

Data presented in this study indicated the need for continuous identification and characterization of MERS-CoV to monitor virus circulation in the region, which is necessary to develop effective control measures. The mutations described in this investigation might not accurately represent the virus’s natural evolution as artificial mutations may develop during cell culture passage. The isolated MERS-CoV strains would be helpful in new live attenuated vaccine development and efficacy studies.

## 1. Introduction

Middle East respiratory syndrome (MERS) is a viral respiratory infection of humans and dromedary camels that is caused by a coronavirus called the Middle East Respiratory Syndrome Coronavirus (MERS-CoV) (1). During the last two decades, the world witnessed the emergence of three novel coronaviruses: severe acute respiratory syndrome coronavirus 1 (SARS CoV-1) in 2002–2003, Middle East respiratory syndrome coronavirus (MERS-CoV) in 2012, and the ongoing pandemic caused by SARS-CoV-2 in 2019, resulting in a very significant impact on both humans and animals as well as the global economy. Coronaviruses can have a devastating impact on the health of humans and animals. Coronaviruses are important RNA viruses, and members of the subfamily Orthocoronavirinae are classified into four genera: Alphacoronavirus, Betacoronavirus, Gammacoronavirus, and Deltacoronavirus (2, 3).

From April 2012 to July 2022, a total of 2,591 laboratory-confirmed cases of Middle East respiratory syndrome (MERS) were reported globally, with 894 associated deaths at a case-fatality ratio (CFR) of 34.5% (4). Via direct or indirect contact, humans can become infected with the MERS-CoV from dromedary camels (*Camelus dromedarius*), the virus’s natural host and zoonotic source. MERS-CoV has been identified in dromedaries in several countries in the Middle East, Africa, and South Asia. Some dromedary camels have shown minor upper respiratory symptoms related to MERS-CoV. While MERS-CoV has little effect on animal health, human infections adversely affect public health. MERS is an emerging disease with severe implications for human public health. MERS-CoV phylogeny currently comprises three major clades, provisionally named clades A, B, and C. The clade B strains currently infect humans and dromedary camels in the Arabian Peninsula, in contrast to clades A and C, which contain extinct strains and strains that are not circulating in this region. Clade B is divided into six phylogenetic lineages. In dromedary camels, the recombination between lineages 3 and 4 led to the emergence of the circulating recombinant lineage 5 around the year 2014 (5–8). More than 41,000 nasal swabs taken from camels in the United Arab Emirates (UAE) between 2013 and 2021 were tested for MERS-CoV by quantitative reverse transcription polymerase chain reaction (RT-qPCR) at the veterinary laboratories of Abu Dhabi Agriculture and Food Safety Authority (ADAFSA). Additionally, we conducted numerous research on various aspects of MERS-CoV infection, including the evaluation of diagnostic tests, epidemiology, and investigation of infection events in humans linked to sick camels, identification of the diversity of the virus in dromedary camels, zoonotic origin and transmission of the virus, and risk factors for the virus seropositivity among animal market and slaughterhouse workers (8–12).

This study aimed to isolate MERS-CoV from nasal swabs of dromedary camels, and propagate and molecularly characterize the isolated virus. The newly isolated virus serves as a good starting point for developing diagnostic tests and inactivated vaccines.

## 2. Materials and methods

### 2.1. Study animals

Dromedary camels were sampled at an open-air animal market in Al Ain, United Arab Emirates for MERS-CoV between September 2016 and November 2017 to investigate risk factors and genetic diversity of the virus. A nasal swab was taken from each of the 372 sampled camels together with information on age, sex, and animal origin. In order to minimize risk when handling MERS-CoV-suspected camel specimens and ensure correct specimen management, prompt communication between ADAFSA collecting veterinarians and laboratory staff was set up.

### 2.2. Sample collection and processing

Nasal samples were obtained by using a Dacron swab kit containing Puritan UniTranz-RT 1 mL Universal Transport Solution (Puritan, Brescia, Italy). All samples were barcoded and then transported to the third-level biological safety laboratory (BSL3) of ADAFSA in Abu Dhabi within 8 h using cool boxes maintained between 4–8°C. Each sample was mixed by pulse-vortexing for 15 s, the solution was transferred to a sterile 5 mL cryovial, and the swab kit was autoclaved and discarded. Samples were centrifuged at 1500 rpm for 10 min at 4°C, and the supernatants were filtered with a 0.45 μ filter (Millipore, Billerica, MA, United States).

### 2.3. Nucleic acid extraction and RT-qPCR

A volume of 400 μL from each nasal swab was used for the nucleic acid extraction with the EZ1 Virus Mini Kit v2.0 (48) kit (Qiagen), and the final elution was done in 60 μL. Screening for MERS-CoV was performed using the Coronavirus MERS-CoV RT-PCR (ModularDx Kit Coronavirus SA1 (EMC) upstream E-gene and Light Mix Modular MERS-CoV Orf1a), targeting the upstream region of the envelope gene (upE) and open reading frame 1a (ORF1a) (13, 14). The RT-qPCR was performed on LightCycler 2.0 using Roche’s LightCycler RNA Virus Master Chemistry (Basel, Switzerland).

### 2.4. Virus isolation

All the nasal swab samples were tested for MERS-CoV by RT-qPCR. Only samples with Cp (cycle threshold) values below 22 were subsequently used for virus isolation. A total of 10 nasal swabs positive by PCR were inoculated into Vero E6 cells (African green monkey kidney) obtained initially from American Type Culture Collection (ATCC, Rockville, MD, United States) specimen CCL-81™. Cells were maintained in Dulbecco’s modified Eagle’s medium with 4.5 g/L D-glucose, and l-glutamine (DMEM, Gibco, Thermo Fisher Scientific, Waltham, MA, United States) supplemented with 10% heat-inactivated fetal bovine serum (Gibco, United States) and 5 mL of Gibco Antibiotic-Antimycotic solution (containing 10,000 units/mL of penicillin, 10,000 μg/mL of streptomycin, and 25 μg/mL of Amphotericin B).

We inoculated Vero E6 cells seeded at 80–90% confluence in 6-well cell culture plates (Corning Inc., United States) with 100 μL of the samples and incubated the cells at 37°C in a 5% carbon dioxide atmosphere. A control well inoculated with sterile phosphate-buffered saline (PBS) was included on each plate. Plates were centrifuged at 1000 rpm (18 × g for 60 min) using a small benchtop centrifuge in a sealed biocontainment bucket (SL 8R Small Benchtop Centrifuge, Thermo Scientific, Germany). This system works because the low-speed centrifugation enhances viral adsorption to the susceptible cells. It is thought that the minor trauma to the cell surface produced because of low-speed centrifugation mechanical force enhances the viral entry into the cells, reducing the total time taken for the virus to produce infection of cells (15).

The inoculum was removed, cells were gently washed three times with DMEM, and a fresh medium was added. The infected cells were monitored daily for 7 days to check for cytopathic effects (CPE) using an inverted microscope, and the medium was changed on alternate days. When 70–90% CPE was observed, the supernatant and cells were harvested by freezing and thawing three times, and confirmation of virus isolation was done by RT-qPCR as described above. For those inoculated samples that showed no CPE, a blind passage was made using the freeze–thaw method. If no CPEs were observed until three successive blind passages, the sample was considered negative. Subsequent passages were performed in T-25 flasks.

### 2.5. Whole genome sequencing

#### 2.5.1. Nucleic acid extraction and sequence-independent, single-primer-amplification

The cell culture extracts (400 μL) of MERS-CoV positive samples were used for the nucleic acid extraction with the EZ1 Virus Mini Kit v2.0 (48) kit (Qiagen). The final elution was done in 60 μL.

Random amplified cDNA was prepared for each sample using an approach that was previously described in another study (16). For reverse transcription, 4 μL of RNA and 1 μL of primer A (5´-GTTTCCCACTGGAGGATA-N9-3′, 40 pmol/μL) were mixed and incubated for 5 min at 65°C followed by cooling to room temperature. To synthesize the first-strand, the volumes of 2 μL SuperScript IV first-strand buffer, 1 μL of 12.5 mM dinucleoside triphosphates (dNTPs), 0.5 μL of 0.1 M dithiothreitol (DTT), 1 μL H_2_O, and 0.5 μL SuperScript IV (Thermo Fisher) were mixed and incubated for 10 min at 42°C. The following substances were mixed to synthesize the second strand: 1 μL Sequenase buffer, 0.85 μL H_2_O, and 0.15 μL Sequenase (Affymetrix) and incubated at 37°C for 8 min, followed by the addition of 0.45 μL Sequenase dilution buffer and 0.15 μL Sequenase. The mixture was incubated for 8 min at 37°C. Amplification of cDNA was performed in triplicate using 5 μL of the reaction mixture as input to a 50 μL of LA Taq (Sigma) reaction mixture, according to the manufacturer’s instructions, using 1 μL primer B (5´-GTTTCCCACTGGAGGATA-3′). The amplification conditions consisted of 98°C for 30 s, and 30 cycles of 94°C for 15 s, 50°C for 20 s, and 68°C for 2 min followed by 68°C for 10 min. The amplified cDNA was purified using AMPure XP beads (Beckman Coulter, Brea, CA) and quantified with a Qubit high-sensitivity double-stranded DNA (dsDNA) kit (Thermo Fisher) following the manufacturer’s instructions.

#### 2.5.2. MiSeq™ library preparation and sequencing

Amplified Round B cDNA from all samples were purified using AMPure XP beads (Beckman Coulter), and 2 ng was used as input into the Nextera XT kit (Illumina) previously described (17). After 13 cycles of amplification, Illumina library concentration and average fragment size were determined using the Agilent 4,200 TapeStation System. Sequencing was performed on an Illumina MiSeq using a V3 MiSeq™ (600 cycles) Reagent kit (Illumina, San Diego, CA, United States).

#### 2.5.3. Long read sequencing

The cDNA of the sample with GenBank ID: OQ423284 (Lab ID: VL_13) and the sample with GenBank ID: OQ423287 (Lab ID: VL_765) were further subjected to long-read sequencing. The library was prepared using Ligation Sequencing Kit (SQK-LSK109), and samples were barcoded with Native Barcoding Expansion 1–12 (EXP-NBD104). Finally, 30 ng of the prepared library was sequenced on a Nanopore MinION device using the R9.4.1 flow cell.

#### 2.5.4. Bioinformatics analysis

The Illumina sequences were first evaluated for their quality using FastQC software,^1^ and the low-quality reads were trimmed with trimmomatic software^2^ using the following settings: TRAILING:28 SLIDINGWINDOW:4:15 MINLEN:35 to remove the reads with quality below 15 or length less than 35 bp. The adaptors were removed from long reads using the Porechop tool.^3^

*De novo* assembly of short reads was performed with the shovill pipeline^4^ with a command: shovill --outdir out --R1 path/to/read1.fasta --R2 path/to/read2.fastq. The accuracy of the assembly was evaluated with the Quast tool^5^ with a command: quast -o assembly/quast path/to/scaffolds.fasta. The hybrid assembly for samples (GenBank IDs: OQ423284 and OQ423287) was accomplished with Spades.^6^ The consensus sequence of the final assembly was first analyzed by BLAST services, followed by phylogenetic analysis. The variant calling against the MERS-CoV reference Saudi isolate JX869059 (EMC/2012) was performed with the snippy tool with an option --mincov 20 (minimum coverage of 20). The nucleotide genome sequences obtained in this study were deposited in GenBank under the accession numbers OQ423283 to OQ423287.

#### 2.5.5. Phylogenetic analysis of MERS-CoV

The BLAST tool available in the NCBI was used for each strain to determine the most closely related sequences of MERS-CoV, and the corresponding sequences, along with the newly isolated sequences, were then used to construct a phylogenetic tree. The final alignment contained 118 sequences from a nearly complete genome (~30,000 nt in length), including human cases of animal origin. The multiple sequence alignment was performed with the ClustalW tool, and the tree was constructed with the Maximum Likelihood method (ML) and Kimura 2-parameter model (18) with 1,000 Bootstrap confidence using MEGA X software (19).

#### 2.5.6. Recombination analysis using RDP5

The Recombination Detection Program (RDP5) v.5.3 (20) was used to detect the possible recombination events using the default settings of algorithms GENECONV, BOOTSCAN, MaxChi, Chimaera, and 3Seq implemented in the RDP5 program. The sequences from this study were aligned with the reference sequences of MERS-CoV representing different clades (~30.000 bp were used for phylogenetic analyses), and the recombination events, likely parental isolates of recombinants, and recombination breakpoints were analyzed.

## 3. Results

### 3.1. Virus isolation

**Figure 1:**
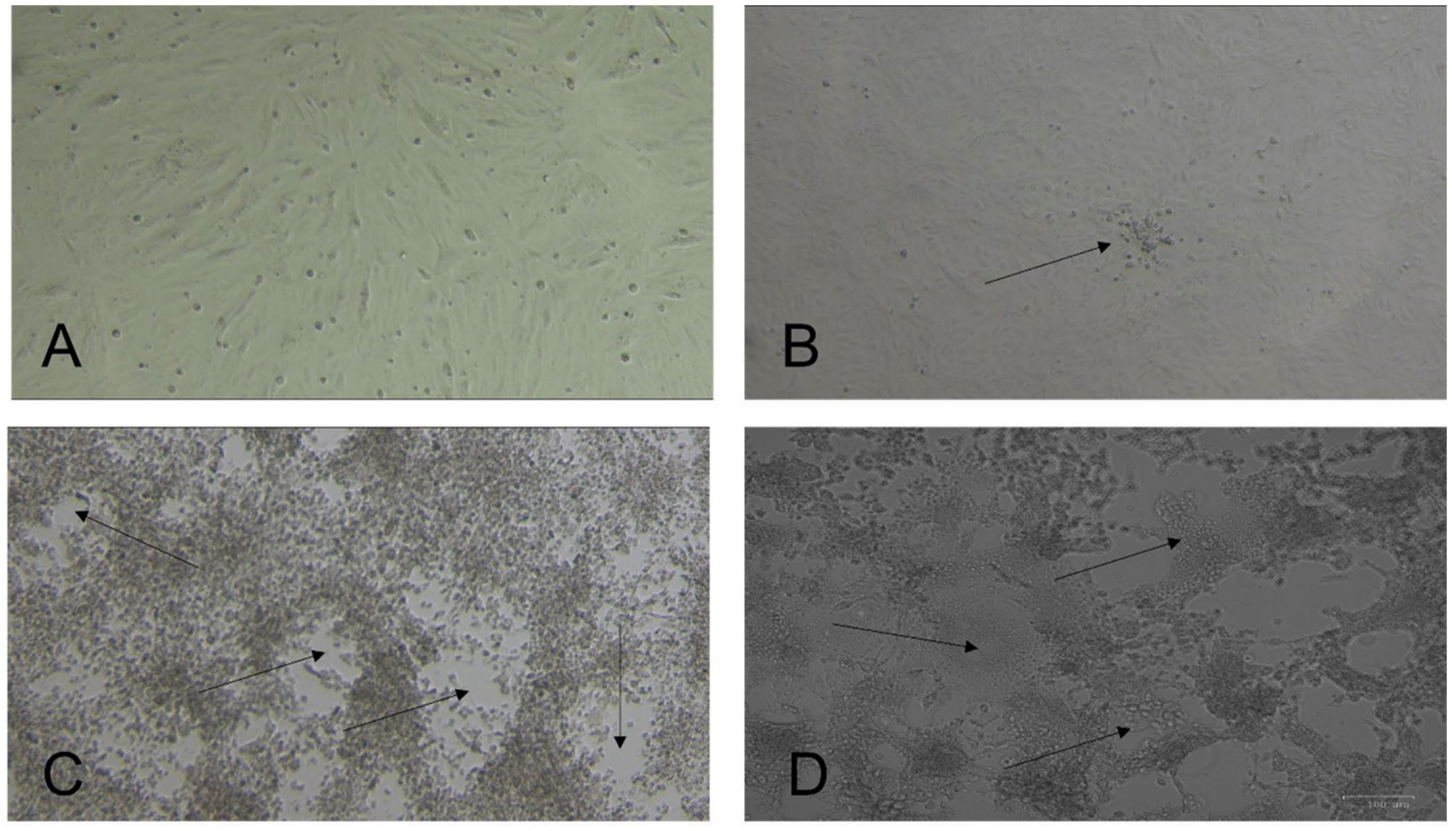
Isolation of Middle East respiratory syndrome coronavirus (MERS-CoV) from nasal swabs of dromedary camels. Vero E6 cells were incubated at 37°C and observed daily for cytopathic effect (CPE) under a light microscope. **(A)** Normal uninfected cells. **(B)** Early CPE of focal cell rounding at day three post-inoculation (PI) (arrow). **(C)** Extensive CPE and plaque formation (arrows) observed on day 5 PI. **(D)** Multinucleated giant cell formation (syncytial formation) (arrows). Original magnification ×10.

After 3 days, we observed virus-induced cytopathic effects (CPE) in three samples, and on the first blind passage, two more isolates were obtained. Table 1 provides information on the five virus isolates and the dromedary camels used for sample collection. Cells infected with all five isolates showed focal rounding and swelling of cells from the fourth day of inoculation, followed by detachment, plaque, and syncytium formation (Figure 1). The presence of MERS-CoV was confirmed by RT-qPCR.

**Table 1:** Details on the five virus isolates and the dromedary camels from which samples were collected.

SN	Animal ID	Virology lab ID	Virus isolates	Date of sample collection	GenBank accession	Animal origin	Camel age (months)	Sex
1	1,060,295	VL-12	MERS-CoV/ dromedary/UAE/Al Ain/12/2016	Oct. 16, 2016	OQ423283	Abu Dhabi	32	Female
2	1,060,297	VL-13	MERS-CoV/ dromedary/UAE/Al Ain /13/2016	Dec. 12, 2016	OQ423284	Al Ain	12	Male
3	10,041,704	VL-704	MERS-CoV/ dromedary/UAE/Al Ain /704/2017	Feb. 2, 2017	OQ423285	Abu Dhabi	12	Male
4	10,041,763	VL-763	MERS-CoV/ dromedary/UAE/Al Ain /763/2017	Sept. 22, 2017	OQ423286	Abu Dhabi	8	Male
5	10,041,765	VL-765	MERS-CoV/dromedary/UAE/ Al Ain/765/2017	Nov. 11, 2017	OQ423287	Al Ain	10	Male

### 3.2. Phylogenetic analysis

**Figure 2:**
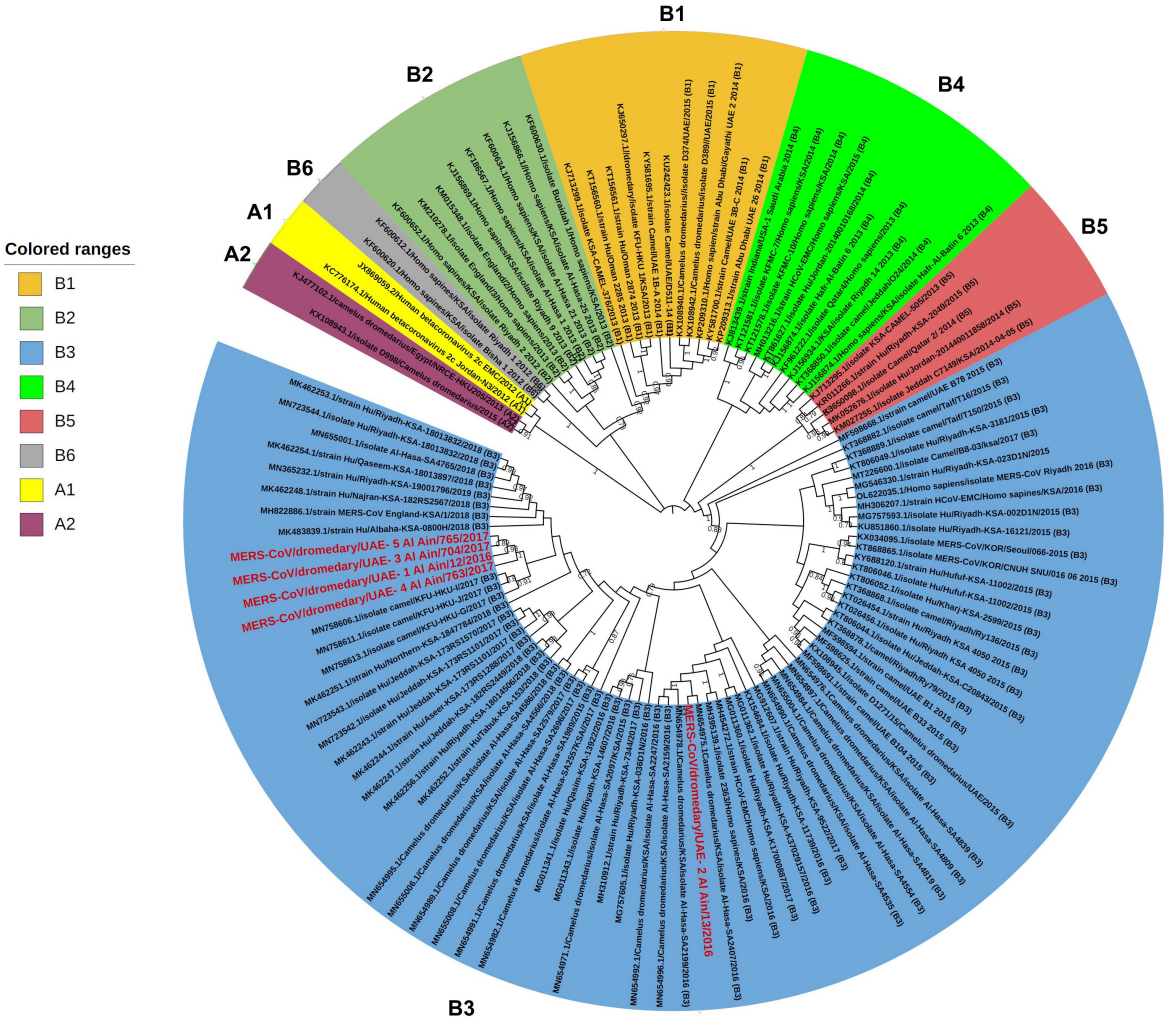
Phylogenetic relationship of MERS-CoV genomes. The tree was constructed based on near-complete MERS-CoV genomes (~30,000 nt in length, *n* = 118), including those of five newly sequenced isolates from the UAE. The tree was constructed with the Maximum Likelihood method and the Kimura 2-parameter model (18) using MEGAX (19). Alignments were performed with ClustalW (21) impeded in MEGAX. Bootstrapping was performed with 1,000 replicates and the value is indicated between 0.7–1 (70–100%). Clade A and clade B are labeled as (A1–A2) and (B1–B6), respectively, while clade C is not shown as it mainly circulates in the African region (22). The sequences obtained in this study are grouped with clade B3 and are marked with the red color. Branch lengths reflect the number of nucleotide substitutions per site.

A phylogenetic analysis using nearly entire genome sequences (30.000 bp) of 118 MERS-CoV sequences, including our isolates, was conducted to determine which group and lineage our isolates belonged to. The result showed that all five isolates clustered within clade B3 with variable relatedness to the previous UAE isolates. The samples with GenBank IDs OQ423283, OQ423285, OQ423286, and OQ423287 formed a separate cluster and were close to the MERS-CoV isolate camel/KFU-HKU-I/2017 (GenBank ID: MN758606.1), as shown in (Figure 2). The sample GenBank ID: OQ423284 was clustered close to the MERS-CoV isolate Al-Hasa-SA2407/2016 (GenBank ID: MN654975.1), as shown in Figure 2.

The MERS-CoV isolates shared 99.69–99.99% nucleotide sequence intragroup identity, 99.73–99.91% identity with the MERS-CoV isolate camel/KFU-HKU-I/2017 (sequence ID: MN758606.1), 99.74–99.86% with the MERS CoV isolate Al-Hasa-SA2407/2016 (GenBank ID: MN654975.1), and 99.42–99.47% identity with the Human betacoronavirus 2c EMC/2012 (GenBank ID: JX869059.2) (Table 2).

**Table 2:** The percent identity matrix created by ClustalW for the MERS-CoV isolates along with close sequence obtained after BLAST analysis.

Strain	OQ423283	OQ423284	OQ423285	OQ423286	OQ423287	MN758606.1	MN654975.1	JX869059
OQ423283/MERS-CoV/dromedary/ UAE-Al Ain/12/2016		99.70%	99.98%	99.96%	99.99%	99.90%	99.74%	99.43%
OQ423284/MERS-CoV/dromedary/ UAE-Al Ain /13/2016	99.70%		99.69%	99.70%	99.70%	99.73%	99.86%	99.47%
OQ423285/MERS-CoV/dromedary/ UAE-Al Ain /704/2017	99.98%	99.69%		99.95%	99.99%	99.90%	99.74%	99.42%
OQ423286/MERS-CoV/dromedary/ UAE-Al Ain /763/2017	99.96%	99.70%	99.95%		99.96%	99.91%	99.74%	99.42%
OQ423287/MERS-CoV/dromedary/UAE-Al Ain/765/2017	99.99%	99.70%	99.99%	99.96%		99.91%	99.75%	99.43%
MN758606.1/isolate camel/KFU-HKU-I/2017	99.90%	99.73%	99.90%	99.91%	99.91%		99.78%	99.46%
MN654975.1/dromedary/KSA/isolate Al-Hasa-SA2407/2016	99.74%	99.86%	99.74%	99.74%	99.75%	99.78%		99.49%
JX869059/Human betacoronavirus 2c EMC/2012	99.43%	99.47%	99.42%	99.42%	99.43%	99.46%	99.49%	

The MERS-CoV reference sequence (JX869059) was also included.

**Table 3:** The missense variants identified from the five isolates (GenBank OQ423283 to OQ423287).

Position in the genome in the reference (JX869059.1)					nt	aa	Gene	Product
OQ423283	OQ423284	OQ423285	OQ423286	OQ423287				
301	301	301	301	301	c.23C > T	**p.Thr8Ile**	orf1ab	ORF1a
750	750	750	750	750	c.472 T > G	**p.Phe158Val**		
1833	1833	1833	1833	1833	c.1555C > A	**p.Leu519Ile**		
2040	2040	2040	2040	2040	c.1762G > A	**p.Ala588Thr**		
2,169	2,169	2,169	2,169	2,169	c.1891A > C	**p.Ile631Leu**		
X	2,456	2,456	X	X	c.2178A > C	p.Lys726Asn		
2,461	2,461	2,461	2,461	2,461	c.2183 T > G	**p.Ile728Ser**		
X	2,938	X	X	X	c.2660C > T	p.Thr887Ile		
X	3,397	X	X	X	c.3119C > T	p.Ala1040Val		
X	3,412	X	X	X	c.3134C > T	p.Ala1045Val		
3,441	3,441	3,441	3,441	3,441	c.3163C > T	**p.Pro1055Ser**		
3,487	3,487	3,487	3,487	3,487	c.3209C > A	**p.Ala1070Glu**		
3,559	3,559	3,559	3,559	3,559	c.3281A > G	**p.Asn1094Ser**		
3,574	X	3,574	3,574	3,574	c.3296C > G	p.Pro1099Arg		
X	3,882	X	X	X	c.3604G > A	p.Val1202Ile		
3,984	3,984	3,984	3,984	3,984	c.3706G > A	**p.Ala1236Thr**		
4,388	4,196	4,388	4,388	4,388	c.4110G > T	**p.Met1370Ile**		
4,401	4,388	4,401	4,401	4,401	c.4123G > A	**p.Val1375Ile**		
4,662	X	4,662	4,662	4,662	c.4384G > A	p.Ala1462Thr		
X	5,139	X	X	X	c.4861C > T	p.His1621Tyr		
5,427	5,427	5,427	5,427	5,427	c.5149 T > A	**p.Leu1717Ile**		
X	5,726	X	X	X	c.5448G > T	p.Glu1816Asp		
X	5,782	X	X	X	c.5504A > C	p.Glu1835Ala		
6,189	X	6,189	X	6,189	c.5911C > T	p.Pro1971Ser		
6,286	6,286	6,286	X	6,286	c.6008C > T	p.Ala2003Val		
6,619	6,619	6,619	6,619	6,619	c.6341C > T	**p.Ala2114Val**		
X	6,635	X	X	X	c.6357G > T	p.Met2119Ile		
X	7,555	X	X	X	c.7277C > T	p.Thr2426Ile		
8,518	8,518	8,518	8,518	8,518	c.8240C > T	**p.Ala2747Val**		
8,617	8,617	8,617	8,617	8,617	c.8339C > T	**p.Ala2780Val**		
9,516	9,516	9,516	9,516	9,516	c.9238A > G	**p.Thr3080Ala**		
13,396	13,396	13,396	13,396	13,396	c.13118C > T	**p.Ala4373Val**		
13,678	13,678	13,678	13,678	13,678	c.13400C > T	**p.Thr4467Ile**	orf1ab	ORF1ab
14,992	14,992	14,992	14,992	14,992	c.14714 T > C	**p.Phe4905Ser**		
15,196	15,196	15,196	15,196	15,196	c.14918C > T	**p.Thr4973Met**		
15,835	15,835	15,835	15,835	15,835	c.15557G > A	**p.Gly5186Asp**		
15,985	15,985	15,985	15,985	15,985	c.15707G > A	**p.Gly5236Asp**		
16,174	16,174	16,174	16,174	16,174	c.15896 T > C	**p.Ile5299Thr**		
16,597	16,597	16,597	16,597	16,597	c.16319C > T	**p.Ser5440Leu**		
16,804	16,804	16,804	16,804	16,804	c.16526 T > C	**p.Phe5509Ser**		
17,089	X	17,089	17,089	17,089	c.16811G > A	p.Gly5604Asp		
17,752	17,752	17,752	17,752	17,752	c.17474C > T	**p.Thr5825Ile**		
17,771	17,771	17,771	17,771	17,771	c.17493 T > G	**p.Cys5831Trp**		
17,794	X	17,794	17,794	17,794	c.17516C > T	p.Pro5839Leu		
17,836	17,836	17,836	17,836	17,836	c.17558C > T	**p.Thr5853Met**		
18,079	18,079	18,079	18,079	18,079	c.17801 T > C	**p.Met5934Thr**		
18,112	X	18,112	18,112	18,112	c.17834 T > C	p.Met5945Thr		
18,146	18,146	18,146	X	18,146	c.17868G > A	p.Met5956Ile		
18,415	18,415	18,415	18,415	18,415	c.18137A > C	**p.His6046Pro**		
19,075	19,075	19,075	19,075	19,075	c.18797G > A	**p.Gly6266Glu**		
19,204	X	19,204	19,204	19,204	c.18926G > A	p.Cys6309Tyr		
19,940	19,940	19,940	19,940	19,940	c.19662 T > G	**p.Ile6554Met**		
19,999	19,999	19,999	19,999	19,999	c.19721C > T	**p.Ser6574Leu**		
20,017	20,017	20,017	20,017	20,017	c.19739G > T	**p.Trp6580Leu**		
20,182	20,182	20,182	20,182	20,182	c.19904 T > G	**p.Phe6635Cys**		
20,848	20,848	20,848	20,848	X	c.20570C > A	p.Pro6857His		
X	X	X	X	20,848	c.20570C > A	p.Pro6857His		
X	22,873	X	X	X	c.1418 T > C	p.Phe473Ser	S	S protein
X	24,514	24,514	X	X	c.3059A > G	p.Gln1020Arg		
25,580	25,580	X	25,580	25,580	c.49C > T	p.Leu17Phe	orf3	ORF3
25,715	25,715	25,715	25,715	25,715	c.184G > T	**p.Val62Phe**		
25,761	25,761	25,761	25,761	25,761	c.230 T > G	**p.Leu77Arg**		
25,788	25,788	25,788	25,788	25,788	c.257C > T	**p.Pro86Leu**		
26,109	26,109	26,109	26,109	26,109	c.17 T > C	**p.Met6Thr**	orf4b	ORF4b
26,167	26,167	26,167	26,167	26,167	c.316C > T	**p.Pro106Ser**		
26,223	26,223	26,223	26,223	26,223	c.131A > C	**p.His44Pro**		
X	26,508	X	X	X	c.416C > T	p.Ala139Val		
28,057	28,057	28,057	28,057	28,057	c.205G > A	p.Val69Ile	M	M protein
X	28,102	X	X	X	c.250G > T	p.Ala84Ser		
28,219	28,219	28,219	28,219	28,219	c.367 T > A	**p.Phe123Ile**		
28,772	28,772	28,772	28,772	28,772	c.11 T > C	**p.Leu4Pro**	orf8b	ORF8b
X	X	X	29,157	X	c.592G > A	p.Gly198Ser	N	N protein

nt, nucleotide; aa, amino acid; and x denotes an absence. Missense variants in bold are shared between the five isolates (GenBank OQ423283 to OQ423287).

### 3.3. Mutational analysis

The single nucleotide polymorphism (SNP) was called based on the original Saudi MERS-CoV reference isolate JXJX869059, where several mutations were reported. Some are unique for these isolates.

#### 3.3.1. Missense variants

In coding region ORF1a, there were 32 missense variants, of which 18/32 were shared between the five isolates, 9/32 were found only in OQ423284, 1/32 was found in samples OQ423284 and OQ423285, 1/32 was found in samples OQ423283, OQ423285 and OQ423287, 2/32 were found in samples OQ423283, OQ423285, OQ423286 and OQ423287, and 1/32 was found in samples OQ423283, OQ423284, OQ423285, and OQ423287. In ORF1ab, there were 25 missense variants, of which 18/25 were shared between the five isolates, 4/25 were found in samples OQ423283, OQ423285, OQ423286 and OQ423287, 1/25 was found in samples OQ423283, OQ423284, OQ423285, and OQ423287, and 1/25 was found in samples OQ423283, OQ423284, OQ423285, and OQ423286. In the spike, there were two missense variants: one variant (p.Phe473Ser) was found in OQ423283 only, while the other (p.Gln1020Arg) was found in samples OQ423283, OQ423284, and OQ423285. In ORF3, there were four missense variants, and three variants (p.Val62Phe, p.Leu77Arg, and p.Pro86Leu) were shared with five samples. Another variant (p.Leu17Phe) was found in samples OQ423283, OQ423284, OQ423286, and OQ423287. In ORF4b, there were four missense variants, of which three variants (p.Met6Thr, p.Pro106Ser, and p.His44Pro) were shared with five samples. Another variant (p.Ala139Val) was found in OQ423283 only. In M, there were three missense variants, of which two variants (p.Val69Ile and p.Phe123Ile) were found in all samples. Another variant (p.Ala84Ser) was found in OQ423284 only. In ORF8b, there was a single missense variant (p.Leu4Pro) detected in all samples. In N, there was a single missense variant (p.Gly198Ser) found in OQ423286 only. The summary of the missense mutations is shown in Table 3 and more details are provided in Supplementary Table S1.

#### 3.3.2. Synonymous variants

In ORF1a, there were 46 synonymous variants, of which 25 were shared between the five isolates. In ORF1ab, there was only one synonymous variant shared between the isolates. There were 17 synonymous variants in S, of which 11 were found in all isolates. In ORF3, there were two synonymous variants. In ORF4a, there were two synonymous variants with one shared between the isolates. In ORF4b, there were four synonymous variants, with only one shared between the isolates. In ORF5, there were five synonymous variants, of which four were shared between the isolates. In M, one synonymous variant was shared between the isolates. In N, there were four synonymous variants. The summary of synonymous variants across the viral genome is provided in Supplementary Table S2.

### 3.4. Analysis of recombination

**Figure 3:**
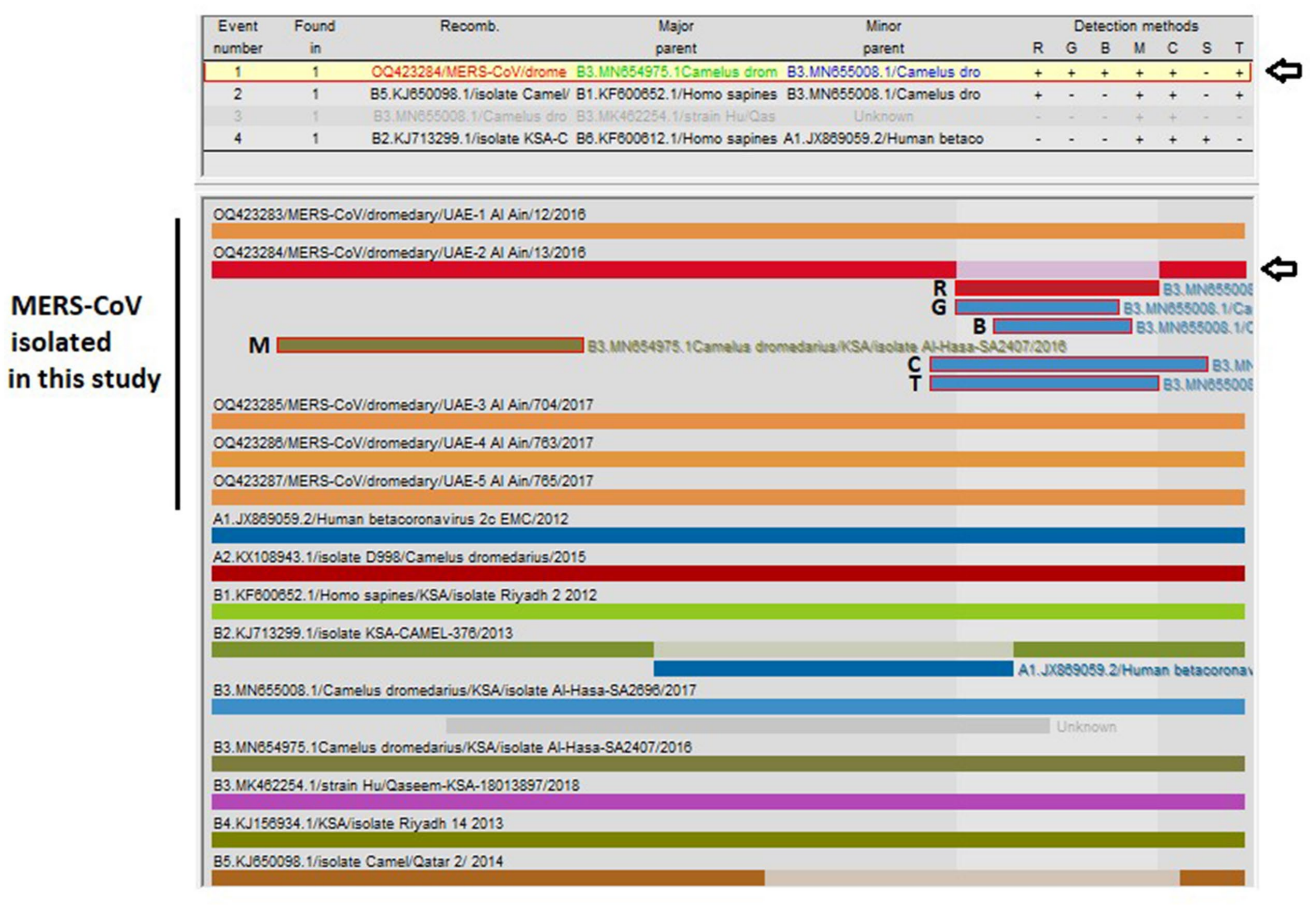
Analysis of possible recombination events in a nearly complete MERS-CoV genome (~30,000 bp). A single recombination event was detected in OQ423284/MERS-CoV/dromedary/UAE-2/Al Ain/13/2016 (indicated by a black arrow). The event detected by the six programs impeded in RDP5 program v.5.3 are indicated (R, RDP; G, GENECONV; B, BootScan; M, MaxiChi; C, Chimaera; T, 3Seq). Detailed information for recombination was also provided in Table 4. Each segment is indicated by a different color bar.

The recombination analysis indicated that OQ423284/MERS-CoV/dromedary/ UAE-Al Ain/13/2016 is a recombinant strain (Figure 3) with close similarity with B3.MN654975.1/*Camelus dromedarius*/KSA/isolate Al-Hasa-SA2407/2016 as a major parent (99.9%) and B3.MN655008.1/*Camelus dromedarius*/KSA/isolate Al-Hasa-SA2696/2017 as a minor parent (99.9%), both of which are nearly identical. The recombination event was detected with six algorithms (RDP, GENECONV, BootScan, MaxiChi, Chimaera, and 3Seq). The beginning and end of the recombination event detected by each algorithm, along with the significant *p*-values, are shown in Table 4.

**Table 4:** Summary of the recombination event identified by the Recombination Detection Program (RDP5) v.5.3.

Recombinant	OQ423284/MERS-CoV/dromedary/ UAE-Al Ain /13/2016	No of sequence detected	Beginning breakpoint (position in the alignment)	Ending breakpoint (position in the alignment)	Av. *p*-value
Major parent	B3.MN654975.1/*Camelus dromedarius*/KSA/isolate Al-Hasa-SA2407//2016				
Minor parent	B3.MN655008.1/*Camelus dromedarius*/KSA/isolate Al-Hasa-SA2696/2017				
*p*-value determined by six different algorithms	RDP	1	20,857 (99.9% similarity)	27,414 (99.9% similarity)	1.271 × 10^04^
	GENECONV	1	21,588 (99.9% similarity)	26,276 (100% similarity)	1.011 × 10^03^
	BootScan	1	22,672 (99.9% similarity)	26,635 (99.9% similarity)	1.304 × 10^02^
	MaxiChi	1	1930 (99.8% similarity)	10,772 (100% similarity)	1.593 × 10^04^
	Chimaera	1	20,857 (99.9% similarity)	28,866 (99.9% similarity)	1.140 × 10^03^
	3Seq	1	20,857 (99.9% similarity)	27,414 (99.9% similarity)	3.834 × 10^07^

## 4. Discussion

MERS-CoV replicates in a wide range of cell types (23, 24) and *in vitro,* and assays for MERS-CoV growth and quantification have been rapidly developed. However, few virus isolates are currently available with full characterization for research work (25–29). Besides, the high mortality rate of ~34% and the continuous introduction of the virus from dromedary camels to humans urge for the establishment of research infrastructure to aid in research and development. Hence, in the current study, attempts were made to isolate the MERS-CoV from nasal swabs of dromedary camels. In the present study, five viral isolates were obtained from nasal swabs of dromedary camels in the UAE and then adapted to *in vitro* cultures and further molecularly characterized to identify strain-specific variations and to determine lineage classification.

The phylogenetic analysis performed in this study indicated that all five isolates belonged to clade B3 of MERS-CoV and were clustered close to the MERS-CoV isolated from camels in Saudi Arabia (KSA) between the years 2016 to 2017. One sample (GenBank ID OQ423284) clustered close to the MERS-CoV detected in the Al Hasa region (GenBank ID: MN654975.1), which was reported in 2016, while other isolates clustered close to the MERS-CoV isolate camel/KFU-HKU-I/2017 (GenBank: MN758606.1), which was reported in 2017.

The clustering of MERS-CoV sequences identified in this study was closely related to camel isolates, which may suggest camel-to-camel transmission and supports camels as an animal reservoir for MERS-CoV. Of note, the sequences of the current isolates were found to cluster distantly in the phylogenetic tree far from the previously known MERS-CoV isolated from camels in the UAE, which were located in lineage 5 and lineage 7 within clade B (8). Therefore, this study may provide insight into the risk of trades and livestock markets in the spread of MERS-CoV between camels.

In many studies, a high frequency of mutations and recombinant events were observed in MERS-CoV camel isolates (30) as well as human isolates, and this is necessary for the virus fitness and adaptation in the host.

In our study, where the virus was sequenced from cell culture-adapted isolates, several mutations were observed. For instance, 32 amino acid substitutions in the ORF1a, 25 amino acid substitutions in the ORF1ab, and a single substitution (p.Gly198Ser) in the N region were reported in samples with GenBank IDs: OQ423285 and OQ423287. The latter mutation was also previously reported in human isolates in KSA (31). Generally, amino acid substitutions in the nsp proteins, as well as the N protein, were found to reduce interaction and subsequent virus replication and progeny production (32).

Moreover, two missense amino acid substitutions in the S protein (p.Phe473Ser in sample 2 and p.Gln1020Arg in samples 2 and 3) were reported. It is known that the p.Phe473Ser is located in the receptor-binding domain (RBD) of the spike gene, and exclusively detected in camel strains in Jeddah and Riyadh (22, 33). The mutation p.Gln1020Arg (Q1020R) is located in the heptad repeat region (HR1) of the spike protein. Other mutations detected in our isolates such as Q1020R, R1020Q, or Q1020H were also reported in camel strains in Egypt, Nigeria, the UAE, and some KSA human strains in Jeddah (34–36); these were found to be associated with major MERS-CoV outbreaks in these countries (22). Therefore, changes in the coronavirus spike should be carefully monitored for their effects on receptor binding, virus transmission (37), or even interspecies jump and spillover to humans (31).

This study also identified three mutations in ORF4b in all isolates and one mutation in samples with GenBank IDs OQ423284 and OQ423287 only. Amino acid substitutions in the ORF4b protein were found to alter the host’s immune response to disease and viral pathogenesis (35, 38).

In addition, one mutation in ORF3 (p.Pro86Leu) observed in all current isolates was also reported previously in MERS-CoV strains in camels from the UAE (39). The biological impact of such amino acid changes in ORF3 (4), M (3), ORF8b (1), and among MERS-CoV strains needs to be fully examined.

Analysis of recombination indicated one of the reported sequences (OQ423284/MERS-CoV/dromedary/UAE-Al Ain/13/2016) is a recombinant strain containing sequence from Al Hasa strains (GenBank ID: MN654975.1 and GenBank ID: MN655008.1) within lineage 3 (Clade B3) with high similarity (99.9%). This is not surprising as recombination frequency due to the exchange of functional motifs or even entire genes is quite high (40) within closely related coronaviruses and between different coronaviruses (3, 41, 42). Recombination events generally affect the evolution and transmission of CoVs, including MERS-CoV, so positive selection sites in the spike protein of MERS-CoV in camels may have enabled virus spillover to humans (6, 26, 43). Therefore, the existence of a recombinant virus in our isolates implies the necessity of continuous surveillance of MERS-CoV infections and variants of concern in camels as well as humans, coupled with efforts toward the development of a MERS vaccine.

Artificial mutations could probably develop during cell culture passage even though the five MERS-CoV isolates analyzed in the current study were from early Vero cell passages and were still regarded as wild-type viruses. The fact that cell culture adaptation causes numerous artificial mutations when SARS-CoV-2 is isolated and multiplied in Vero-related cells is well documented (44). The recovery of MERS-CoV from synthesized RNA via the Baric approach in Vero cells is known to easily cause the T1015N artificial mutation in the S protein (45). Additionally, the repetitive passage of MERS-CoV on BHK cells that express DPP4 results in tailored mutations in the spike protein, even if Vero cells are not involved (46). Accordingly, the mutations described in this investigation might not accurately represent the virus’s natural evolution. It is recommended that directly sequencing the wild-type virus from the positive samples using the amplicon method (36) or the SISPA method (16) is the next step to validate mutations reported in this study.

## 5. Conclusion

In conclusion, MERS-CoV genomic sequences determined in this study are similar to those of viruses detected in camels in Saudi Arabia during the period 2016–2018. Phylogenetic analysis of MERS-CoV isolates indicated the virus lineage 3 clade B viruses continue to be dominant among camels in the UAE. Sequence analysis identified several mutations in different virus proteins as well as a recombination event, raising concerns regarding new viral outbreaks and disease severity. The results of this study support the necessity for the complete genome sequencing of all new cases of MERS-CoV to monitor virus circulation in the region and to develop effective control measures. Although the mutations detected in this study may not reflect the true natural evolution of the virus, as artificial mutations may occur during cell culture passage, the isolated MERS-CoV would be useful in new vaccine development and efficacy studies, pathogenicity, and antiviral research.

## Data availability statement

The datasets presented in this study can be found in online repositories. The names of the repository/repositories and accession number(s) can be found in the article/Supplementary material.

## Ethics statement

The current study involving swab samples from dromedary camels was reviewed and approved by the ADAFSA Ethics Committee for Monitoring the use of Animals in Scientific Research (ADAFSA-EA-08-2016) and the use of these samples for virus isolation was permitted without additional ethical approval. The study was conducted in accordance with the local legislation and institutional requirements.

## Author contributions

AK and HI: conceptualization and data analysis. AK, HI, and SM: methodology, data curation, and writing-original draft preparation. HA and ZA-H: sample preparation and testing. AK, HI, and AS: writing-review and editing. AS and SM: supervision and funding acquisition. All authors contributed to the article and approved the submitted version.

## Funding

This work was funded by Abu Dhabi Agriculture and Food Safety Authority (ADAFSA), Abu Dhabi, UAE.

## Conflict of interest

The authors declare that the research was conducted in the absence of any commercial or financial relationships that could be construed as a potential conflict of interest.

## Publisher’s note

All claims expressed in this article are solely those of the authors and do not necessarily represent those of their affiliated organizations, or those of the publisher, the editors and the reviewers. Any product that may be evaluated in this article, or claim that may be made by its manufacturer, is not guaranteed or endorsed by the publisher.

## Footnotes

^1^https://github.com/s-andrews/FastQC

^2^https://github.com/usadellab/Trimmomatic

^3^https://github.com/rrwick/Porechop

^4^https://github.com/tseemann/shovill

^5^https://github.com/ablab/quast

^6^https://github.com/ablab/spades
