# Supplementary material for: Isolation and genetic characterization of MERS-CoV from dromedary camels in the United Arab Emirates
Source: Front Vet Sci. 2023 Aug 31;10:1182165. doi: 10.3389/fvets.2023.1182165 (PMC10500840; doi:10.3389/fvets.2023.1182165)
Supplement: Supplementary file 1 [file Table_1.docx]

**Supplementary Table 1.** The missense variants identified from the isolates.

| **Position in the Chromosome (GenBank ID:JX869059)** | | | | | **NT** | **AA** | **NT_POS** | **AA_POS** | **GENE** |
| --- | --- | --- | --- | --- | --- | --- | --- | --- | --- |
| **OQ423283** | **OQ423284** | **OQ423285** | **OQ423286** | **OQ423287** |  |  |  |  |  |
| 301 | 301 | 301 | 301 | 301 | c.23C>T | **p.Thr8Ile** | 23/13176 | 8/4391 | **orf1ab** |
| 750 | 750 | 750 | 750 | 750 | c.472T>G | **p.Phe158Val** | 472/13176 | 158/4391 |  |
| 1833 | 1833 | 1833 | 1833 | 1833 | c.1555C>A | **p.Leu519Ile** | 1555/13176 | 519/4391 |  |
| 2040 | 2040 | 2040 | 2040 | 2040 | c.1762G>A | **p.Ala588Thr** | 1762/13176 | 588/4391 |  |
| 2169 | 2169 | 2169 | 2169 | 2169 | c.1891A>C | **p.Ile631Leu** | 1891/13176 | 631/4391 |  |
| x | 2456 | 2456 | x | x | c.2178A>C | p.Lys726Asn | 2178/13176 | 726/4391 |  |
| 2461 | 2461 | 2461 | 2461 | 2461 | c.2183T>G | **p.Ile728Ser** | 2183/13176 | 728/4391 |  |
| x | 2938 | x | x | x | c.2660C>T | p.Thr887Ile | 2660/13176 | 887/4391 |  |
| x | 3397 | x | x | x | c.3119C>T | p.Ala1040Val | 3119/13176 | 1040/4391 |  |
| x | 3412 | x | x | x | c.3134C>T | p.Ala1045Val | 3134/13176 | 1045/4391 |  |
| 3441 | 3441 | 3441 | 3441 | 3441 | c.3163C>T | **p.Pro1055Ser** | 3163/13176 | 1055/4391 |  |
| 3487 | 3487 | 3487 | 3487 | 3487 | c.3209C>A | **p.Ala1070Glu** | 3209/13176 | 1070/4391 |  |
| 3559 | 3559 | 3559 | 3559 | 3559 | c.3281A>G | **p.Asn1094Ser** | 3281/13176 | 1094/4391 |  |
| 3574 | x | 3574 | 3574 | 3574 | c.3296C>G | p.Pro1099Arg | 3296/13176 | 1099/4391 |  |
| x | 3882 | x | x | x | c.3604G>A | p.Val1202Ile | 3604/13176 | 1202/4391 |  |
| 3984 | 3984 | 3984 | 3984 | 3984 | c.3706G>A | **p.Ala1236Thr** | 3706/13176 | 1236/4391 |  |
| 4388 | 4196 | 4388 | 4388 | 4388 | c.4110G>T | **p.Met1370Ile** | 4110/13176 | 1370/4391 |  |
| 4401 | 4388 | 4401 | 4401 | 4401 | c.4123G>A | **p.Val1375Ile** | 4123/13176 | 1375/4391 |  |
| 4662 | x | 4662 | 4662 | 4662 | c.4384G>A | p.Ala1462Thr | 4384/13176 | 1462/4391 |  |
| x | 5139 | x | x | x | c.4861C>T | p.His1621Tyr | 4861/13176 | 1621/4391 |  |
| 5427 | 5427 | 5427 | 5427 | 5427 | c.5149T>A | **p.Leu1717Ile** | 5149/13176 | 1717/4391 |  |
| x | 5726 | x | x | x | c.5448G>T | p.Glu1816Asp | 5448/13176 | 1816/4391 |  |
| x | 5782 | x | x | x | c.5504A>C | p.Glu1835Ala | 5504/13176 | 1835/4391 |  |
| 6189 | x | 6189 | x | 6189 | c.5911C>T | p.Pro1971Ser | 5911/13176 | 1971/4391 |  |
| 6286 | 6286 | 6286 | x | 6286 | c.6008C>T | p.Ala2003Val | 6008/13176 | 2003/4391 |  |
| 6619 | 6619 | 6619 | 6619 | 6619 | c.6341C>T | **p.Ala2114Val** | 6341/13176 | 2114/4391 |  |
| x | 6635 | x | x | x | c.6357G>T | p.Met2119Ile | 6357/13176 | 2119/4391 |  |
| x | 7555 | x | x | x | c.7277C>T | p.Thr2426Ile | 7277/13176 | 2426/4391 |  |
| 8518 | 8518 | 8518 | 8518 | 8518 | c.8240C>T | **p.Ala2747Val** | 8240/13176 | 2747/4391 |  |
| 8617 | 8617 | 8617 | 8617 | 8617 | c.8339C>T | **p.Ala2780Val** | 8339/13176 | 2780/4391 |  |
| 9516 | 9516 | 9516 | 9516 | 9516 | c.9238A>G | **p.Thr3080Ala** | 9238/13176 | 3080/4391 |  |
| 13396 | 13396 | 13396 | 13396 | 13396 | c.13118C>T | **p.Ala4373Val** | 13118/13176 | 4373/4391 |  |
| 13678 | 13678 | 13678 | 13678 | 13678 | c.13400C>T | **p.Thr4467Ile** | 13400/21236 | 4467/7077 | **orf1ab** |
| 14992 | 14992 | 14992 | 14992 | 14992 | c.14714T>C | **p.Phe4905Ser** | 14714/21236 | 4905/7077 |  |
| 15196 | 15196 | 15196 | 15196 | 15196 | c.14918C>T | **p.Thr4973Met** | 14918/21236 | 4973/7077 |  |
| 15835 | 15835 | 15835 | 15835 | 15835 | c.15557G>A | **p.Gly5186Asp** | 15557/21236 | 5186/7077 |  |
| 15985 | 15985 | 15985 | 15985 | 15985 | c.15707G>A | **p.Gly5236Asp** | 15707/21236 | 5236/7077 |  |
| 16174 | 16174 | 16174 | 16174 | 16174 | c.15896T>C | **p.Ile5299Thr** | 15896/21236 | 5299/7077 |  |
| 16597 | 16597 | 16597 | 16597 | 16597 | c.16319C>T | **p.Ser5440Leu** | 16319/21236 | 5440/7077 |  |
| 16804 | 16804 | 16804 | 16804 | 16804 | c.16526T>C | **p.Phe5509Ser** | 16526/21236 | 5509/7077 |  |
| 17089 | x | 17089 | 17089 | 17089 | c.16811G>A | p.Gly5604Asp | 16811/21236 | 5604/7077 |  |
| 17752 | 17752 | 17752 | 17752 | 17752 | c.17474C>T | **p.Thr5825Ile** | 17474/21236 | 5825/7077 |  |
| 17771 | 17771 | 17771 | 17771 | 17771 | c.17493T>G | **p.Cys5831Trp** | 17493/21236 | 5831/7077 |  |
| 17794 | x | 17794 | 17794 | 17794 | c.17516C>T | p.Pro5839Leu | 17516/21236 | 5839/7077 |  |
| 17836 | 17836 | 17836 | 17836 | 17836 | c.17558C>T | **p.Thr5853Met** | 17558/21236 | 5853/7077 |  |
| 18079 | 18079 | 18079 | 18079 | 18079 | c.17801T>C | **p.Met5934Thr** | 17801/21236 | 5934/7077 |  |
| 18112 | x | 18112 | 18112 | 18112 | c.17834T>C | p.Met5945Thr | 17834/21236 | 5945/7077 |  |
| 18146 | 18146 | 18146 | x | 18146 | c.17868G>A | p.Met5956Ile | 17868/21236 | 5956/7077 |  |
| 18415 | 18415 | 18415 | 18415 | 18415 | c.18137A>C | **p.His6046Pro** | 18137/21236 | 6046/7077 |  |
| 19075 | 19075 | 19075 | 19075 | 19075 | c.18797G>A | **p.Gly6266Glu** | 18797/21236 | 6266/7077 |  |
| 19204 | x | 19204 | 19204 | 19204 | c.18926G>A | p.Cys6309Tyr | 18926/21236 | 6309/7077 |  |
| 19940 | 19940 | 19940 | 19940 | 19940 | c.19662T>G | **p.Ile6554Met** | 19662/21236 | 6554/7077 |  |
| 19999 | 19999 | 19999 | 19999 | 19999 | c.19721C>T | **p.Ser6574Leu** | 19721/21236 | 6574/7077 |  |
| 20017 | 20017 | 20017 | 20017 | 20017 | c.19739G>T | **p.Trp6580Leu** | 19739/21236 | 6580/7077 |  |
| 20182 | 20182 | 20182 | 20182 | 20182 | c.19904T>G | **p.Phe6635Cys** | 19904/21236 | 6635/7077 |  |
| 20848 | 20848 | 20848 | 20848 | x | c.20570C>A | p.Pro6857His | 20570/21236 | 6857/7077 |  |
| x | x | x | x | 20848 | c.20570C>A | p.Pro6857His | 20570/21236 | 6857/7077 |  |
| x | 22873 | x | x | x | c.1418T>C | p.Phe473Ser | 1418/4062 | 473/1353 | **S** |
| x | 24514 | 24514 | x | x | c.3059A>G | p.Gln1020Arg | 3059/4062 | 1020/1353 |  |
| 25580 | 25580 | x | 25580 | 25580 | c.49C>T | p.Leu17Phe | 49/312 | 17/103 | **orf3** |
| 25715 | 25715 | 25715 | 25715 | 25715 | c.184G>T | **p.Val62Phe** | 184/312 | 62/103 |  |
| 25761 | 25761 | 25761 | 25761 | 25761 | c.230T>G | **p.Leu77Arg** | 230/312 | 77/103 |  |
| 25788 | 25788 | 25788 | 25788 | 25788 | c.257C>T | **p.Pro86Leu** | 257/312 | 86/103 |  |
| 26109 | 26109 | 26109 | 26109 | 26109 | c.17T>C | **p.Met6Thr** | 17/741 | 6/246 | **orf4b** |
| 26167 | 26167 | 26167 | 26167 | 26167 | c.316C>T | **p.Pro106Ser** | 316/330 | 106/109 |  |
| 26223 | 26223 | 26223 | 26223 | 26223 | c.131A>C | **p.His44Pro** | 131/741 | 44/246 |  |
| x | 26508 | x | x | x | c.416C>T | p.Ala139Val | 416/741 | 139/246 |  |
| 28057 | 28057 | 28057 | 28057 | 28057 | c.205G>A | p.Val69Ile | 205/660 | 69/219 | **M** |
| x | 28102 | x | x | x | c.250G>T | p.Ala84Ser | 250/660 | 84/219 |  |
| 28219 | 28219 | 28219 | 28219 | 28219 | c.367T>A | **p.Phe123Ile** | 367/660 | 123/219 |  |
| 28772 | 28772 | 28772 | 28772 | 28772 | c.11T>C | **p.Leu4Pro** | 11/339 | 4/112 | **orf8b** |
| x | x | x | 29157 | x | c.592G>A | p.Gly198Ser | 592/1242 | 198/413 | **N** |

NT = nucleotide position of the variant and AA = amino acids position of the synonymous variant. Bold are synonymous variant shared between the five isolates (GenBank OQ423283 to OQ423287).

**Supplementary Table 2.** The synonymous variants identified from the isolates.

| **Position in the Chromosome (GenBank ID:JX869059)** | | | | | **NT** | **AA** | **NT_POS** | **AA_POS** | **GENE** |
| --- | --- | --- | --- | --- | --- | --- | --- | --- | --- |
| **OQ423283** | **OQ423284** | **OQ423285** | **OQ423286** | **OQ423287** |  |  |  |  |  |
| 476 | 476 | 476 | 476 | 476 | c.198G>A | **p.Lys66Lys** | 198/13176 | 66/4391 | **orf1ab** |
| x | 1262 | x | x | x | c.984C>T | p.Thr328Thr | 984/13176 | 328/4391 |  |
| x | 1454 | x | 1454 | x | c.1176T>C | p.Asp392Asp | 1176/13176 | 392/4391 |  |
| 2009 | 2009 | 2009 | 2009 | 2009 | c.1731T>C | **p.Tyr577Tyr** | 1731/13176 | 577/4391 |  |
| 2318 | 2318 | 2318 | 2318 | 2318 | t c.2040C>T | **p.Leu680Leu** | 2040/13176 | 680/4391 |  |
| x | 2393 | x | x | x | c. 2115G>T | p.Val705Val | 2115/13176 | 705/4391 |  |
| x | 2696 | x | x | x | c.2418T>C | p.His806His | 2418/13176 | 806/4391 |  |
| 2774 | 2774 | 2774 | 2774 | 2774 | c.2496T>C | **p.Phe832Phe** | 2496/13176 | 832/4391 |  |
| 3134 | 3134 | 3134 | 3134 | 3134 | c.2856C>T | **p.Ser952Ser** | 2856/13176 | 952/4391 |  |
| 3320 | 3320 | 3320 | 3320 | 3320 | c.3042T>C | **p.Pro1014Pro** | 3042/13176 | 1014/4391 |  |
| 3431 | x | 3431 | 3431 | 3431 | t c.3153T>C | p.Thr1051Thr | 3153/13176 | 1051/4391 |  |
| 3785 | x | 3785 | 3785 | 3785 | c.3507A>G | p.Gln1169Gln | 3507/13176 | 1169/4391 |  |
| x | 3845 | x | x | x | c.3567C>T | p.Leu1189Leu | 3567/13176 | 1189/4391 |  |
| 4034 | 4034 | 4034 | 4034 | 4034 | c.3756G>A | **p.Arg1252Arg** | 3756/13176 | 1252/4391 |  |
| 4514 | x | 4514 | 4514 | 4514 | c.4236C>T | p.His1412His | 4236/13176 | 1412/4391 |  |
| 4847 | 4847 | 4847 | 4847 | 4847 | c.4569C>T | **p.Asp1523Asp** | 4569/13176 | 1523/4391 |  |
| 5516 | 5516 | 5516 | 5516 | 5516 | c.5238A>G | **p.Ala1746Ala** | 5238/13176 | 1746/4391 |  |
| 6059 | 6059 | 6059 | 6059 | 6059 | c.5781T>C | **p.Asn1927Asn** | 5781/13176 | 1927/4391 |  |
| 6293 | 6293 | 6293 | 6293 | 6293 | c.6015T>C | **p.Asp2005Asp** | 6015/13176 | 2005/4391 |  |
| 6332 | 6332 | 6332 | 6332 | 6332 | c.6054C>T | **p.Asp2018Asp** | 6054/13176 | 2018/4391 |  |
| 6518 | x | 6518 | 6518 | x | c.6240C>T | p.Thr2080Thr | 6240/13176 | 2080/4391 |  |
| x | x | x | x | 6518 | c.6240C>T | p.Thr2080Thr | 6240/13176 | 2080/4391 |  |
| 6707 | 6707 | 6707 | 6707 | 6707 | c.6429C> | **T p.Ala2143Ala** | 6429/13176 | 2143/4391 |  |
| 7001 | 7001 | 7001 | 7001 | 7001 | c.6723C>A | **p.Ser2241Ser** | 6723/13176 | 2241/4391 |  |
| x | 7070 | x | x | x | c.6792C>T | p.Ser2264Ser | 6792/13176 | 2264/4391 |  |
| x | 7877 | x | x | x | c.7599T>C | p.Tyr2533Tyr | 7599/13176 | 2533/4391 |  |
| 7973 | x | 7973 | 7973 | 7973 | c.7695T>C | p.Asp2565Asp | 7695/13176 | 2565/4391 |  |
| 8207 | 8207 | 8207 | 8207 | 8207 | c.7929C>T | **p.Ser2643Ser** | 7929/13176 | 2643/4391 |  |
| 8258 | 8258 | 8258 | 8258 | 8258 | c.7980C>T | **p.Ser2660Ser** | 7980/13176 | 2660/4391 |  |
| 8333 | 8333 | 8333 | 8333 | 8333 | c.8055C>T | **p.Cys2685Cys** | 8055/13176 | 2685/4391 |  |
| 8546 | 8546 | 8546 | 8546 | 8546 | c.8268T>C | **p.Tyr2756Tyr** | 8268/13176 | 2756/4391 |  |
| 9395 | x | 9395 | 9395 | 9395 | c.9117G>A | p.Ala3039Ala | 9117/13176 | 3039/4391 |  |
| x | 9413 | x | x | x | c.9135C>T | p.Phe3045Phe | 9135/13176 | 3045/4391 |  |
| 9740 | x | 9740 | 9740 | 9740 | c.9462T>C | p.Asp3154Asp | 9462/13176 | 3154/4391 |  |
| 10505 | 10505 | 10505 | 10505 | 10505 | c.10227C>T | **p.Phe3409Phe** | 10227/13176 | 3409/4391 |  |
| x | 10511 | x | x | x | c.10233C>T | p.Tyr3411Tyr | 10233/13176 | 3411/4391 |  |
| 10835 | 10835 | 10835 | 10835 | 10835 | c.10557G>T | **p.Leu3519Leu** | 10557/13176 | 3519/4391 |  |
| 10874 | 10874 | 10874 | 10874 | 10874 | c.10596C>T | **p.Thr3532Thr** | 10596/13176 | 3532/4391 |  |
| x | x | x | 10982 | x | c.10704T>C | p.Phe3568Phe | 10704/13176 | 3568/4391 |  |
| x | 11447 | x | x | x | c.11169C>T | p.Val3723Val | 11169/13176 | 3723/4391 |  |
| 11984 | 11984 | 11984 | 11984 | 11984 | c.11706T>C | **p.Ala3902Ala** | 11706/13176 | 3902/4391 |  |
| 12425 | 12425 | 12425 | 12425 | 12425 | c.12147C>T | **p.Ile4049Ile** | 12147/13176 | 4049/4391 |  |
| 12684 | 12684 | 12684 | 12684 | 12684 | c.12406C>T | **p.Leu4136Leu** | 12406/13176 | 4136/4391 |  |
| 12707 | 12707 | 12707 | x | 12707 | c.12429G>A | p.Ala4143Ala | 12429/13176 | 4143/4391 |  |
| 12845 | 12845 | 12845 | 12845 | 12845 | c.12567C>T | **p.Val4189Val** | 12567/13176 | 4189/4391 |  |
| 13022 | 13022 | 13022 | 13022 | 13022 | c.12744C>T | **p.Ser4248Ser** | 12744/13176 | 4248/4391 |  |
| 14162 | 14162 | 14162 | 14162 | 14162 | c.13884C>T | **p.Val4628Val** | 13884/21236 | 4628/7077 | **orf1ab** |
| 21713 | x | 21713 | 21713 | 21713 | c.258T>C | p.Val86Val | 258/4062 | 86/1353 | **S** |
| 22742 | x | x | 22742 | 22742 | c.1287T>C | p.Ser429Ser | 1287/4062 | 429/1353 |  |
| 22790 | 22790 | 22790 | 22790 | 22790 | c.1335C>T | **p.Tyr445Tyr** | 1335/4062 | 445/1353 |  |
| 22871 | x | 22871 | 22871 | 22871 | c.1416C>T | p.Ser472Ser | 1416/4062 | 472/1353 |  |
| 22886 | x | 22886 | 22886 | 22886 | c.1431A>T | p.Thr477Thr | 1431/4062 | 477/1353 |  |
| 23504 | 23504 | 23504 | 23504 | 23504 | c.2049T>C | **p.Ser683Ser** | 2049/4062 | 683/1353 |  |
| 23570 | 23570 | 23570 | 23570 | 23570 | c.2115C>T | **p.Gly705Gly** | 2115/4062 | 705/1353 |  |
| 23804 | 23804 | 23804 | 23804 | 23804 | c.2349C>T | **p.Pro783Pro** | 2349/4062 | 783/1353 |  |
| 24191 | 24191 | 24191 | 24191 | 24191 | c.2736C>T | **p.Cys912Cys** | 2736/4062 | 912/1353 |  |
| 24251 | 24251 | 24251 | 24251 | 24251 | c.2796C>T | **p.Tyr932Tyr** | 2796/4062 | 932/1353 |  |
| x | 24296 | 24296 | x | x | c.2841T>C | p.Tyr947Tyr | 2841/4062 | 947/1353 |  |
| 24698 | 24698 | 24698 | 24698 | 24698 | c.3243T>C | **p.Phe1081Phe** | 3243/4062 | 1081/1353 |  |
| x | 24791 | x | x | x | c.3336G>A | p.Lys1112Lys | 3336/4062 | 1112/1353 |  |
| 24863 | 24863 | 24863 | 24863 | 24863 | c.3408C>T | **p.Phe1136Phe** | 3408/4062 | 1136/1353 |  |
| 25046 | 25046 | 25046 | 25046 | 25046 | c.3591T>C | **p.Ile1197Ile** | 3591/4062 | 1197/1353 |  |
| 25052 | 25052 | 25052 | 25052 | 25052 | c.3597C>T | **p.Ser1199Ser** | 3597/4062 | 1199/1353 |  |
| 25208 | 25208 | 25208 | 25208 | 25208 | c.3753C>T | **p.Ser1251Ser** | 3753/4062 | 1251/1353 |  |
| 25570 | 25570 | 25570 | 25570 | 25570 | c.39T>C | **p.Phe13Phe** | 39/312 | 13/103 | **orf3** |
| x | 25768 | x | 25768 | x | c.237A>T | p.Ser79Ser | 237/312 | 79/103 |  |
| 25899 | 25899 | 25899 | 25899 | 25899 | c.48C>T | **p.Asn16Asn** | 48/330 | 16/109 | **orf4a** |
| 25923 | 25923 | 25923 | 25923 | 25923 | c.72C>T | **p.Tyr24Tyr** | 72/330 | 24/109 |  |
| 26665 | x | 26665 | 26665 | 26665 | c.573G>T | p.Leu191Leu | 573/741 | 191/246 | **orf4b** |
| 26716 | x | 26716 | 26716 | 26716 | c.624C>T | p.Tyr208Tyr | 624/741 | 208/246 |  |
| x | 26755 | x | x | x | c.663T>C | p.Val221Val | 663/741 | 221/246 |  |
| 26806 | 26806 | 26806 | 26806 | 26806 | c.714T>C | **p.Tyr238Tyr** | 714/741 | 238/246 |  |
| 26926 | x | 26926 | 26926 | 26926 | c.87T>C | p.Ile29Ile | 87/675 | 29/224 | **orf5** |
| 27067 | 27067 | 27067 | 27067 | 27067 | c.228G>T | **p.Leu76Leu** | 228/675 | 76/224 |  |
| 27229 | 27229 | 27149 | 27229 | 27229 | c.390C>T | **p.Ser130Ser** | 390/675 | 130/224 |  |
| 27277 | 27277 | 27277 | 27277 | 27277 | c.438A>C | **p.Val146Val** | 438/675 | 146/224 |  |
| 27355 | 27355 | 27355 | 27355 | 27355 | c.516C>T | **p.Ser172Ser** | 516/675 | 172/224 |  |
| 28290 | 28290 | 28290 | 28290 | 28290 | c.438C>T | **p.Gly146Gly** | 438/660 | 146/219 | **M** |
| 28634 | x | 28634 | x | 28634 | c.69A>G | p.Leu23Leu | 69/1242 | 23/413 | **N** |
| x | 28715 | x | x | x | c.150C>T | p.His50His | 150/1242 | 50/413 |  |
| x | x | x | 29202 | x | c.637C>T | p.Leu213Leu | 637/1242 | 213/413 |  |
| 29657 | x | 29657 | 29657 | 29657 | c.1092G>A | p.Lys364Lys | 1092/1242 | 364/413 |  |

NT = nucleotide position of the variant and AA = amino acids position of the synonymous variant. Bold are synonymous variant shared between the five isolates (GenBank OQ423283 to OQ423287).
